# Evaluation of *Rumex hastatus* D. Don for cytotoxic potential against HeLa and NIH/3T3 cell lines: chemical characterization of chloroform fraction and identification of bioactive compounds

**DOI:** 10.1186/s12906-016-1302-y

**Published:** 2016-08-24

**Authors:** Sajjad Ahmad, Farhat Ullah, Anwar Zeb, Muhammad Ayaz, Farman Ullah, Abdul Sadiq

**Affiliations:** 1 Department of Pharmacy, University of Malakand, Dir (L), Chakdara, 18000 KPK Pakistan; 2 Department of Pharmacy, Kohat University of Science & Technology, 26000 Kohat, KPK Pakistan

**Keywords:** *Rumex hastatus*, Cytotoxicity, Anticancer, HeLa, NIH/3T3, GC-MS

## Abstract

**Background:**

The importance of Rumex genus and the renowned ethnopharmacological and biological potentials of *Rumex hastatus* is evident from the previous reports. Recently the *R. hastatus* has been evaluated for anticancer potential against HepG2, MCF7 or LNCaP cell lines with considerable cytotoxicity. We also reported the anti-tumor and anti-angiogenic potentials of *R. hastatus.* The current study has been arranged to evaluate cytotoxic potential of this plant against HeLa and NIH/3T3 cell lines and sort out the most active fraction of *R. hastatus* along with the identification of bioactive compounds responsible for cytotoxicity.

**Methods:**

The cytotoxic potential of methanolic extract and sub-fractions of *R. hastatus* was performed following (3-[4, 5-dimethylthiazole-2-yl]-2, 5-diphenyl-tetrazolium bromide) MTT calorimetric assay. Four concentrations (500, 250, 125 and 62.5 μg/ml) of each sample were used against both cell lines. Two cell lines i.e. HeLa and NIH/3T3 were used in the assay. Furthermore, chemical characterization of chloroform fraction was performed by GC-MS analysis.

**Results:**

The current investigational study demonstrates that all the solvent fractions of *R. hastatus* were active against HeLa and NIH/3T3 cell lines. Among all the fractions, chloroform fraction was dominant in activity against both cell lines. The observed IC_50_ values of chloroform fraction were 151.52 and 53.37 μg/ml against HeLa and NIH/3T3 respectively. The GC-MS analysis of chloroform fraction revealed the identification of 78 compounds with the identification of bioactive ones like ar-tumerone, phytol, dihydrojasmone, sitostenone etc.

**Conclusion:**

It can be concluded from our results that *Rumex hastatus* D. Don possess strong cytotoxic potential. Moreover, the observed IC_50_ values and GC-MS analysis of chloroform fraction reveal that most of the bioactive compounds are in chloroform fraction. It can be further deduce that the chloroform fraction is a suitable target for the isolation of compounds having potential role in cancer therapy.

## Background

The leading research teams around the world are in continuous struggle to explore novel aspects to facilitate life. The facilitation of life also encompasses decreased morbidity and mortality [1]. One of leading causes of mortality is cancer worldwide which is considered as the most challenging disease. Several factors have been reported which cause cancer and hyper proliferative conditions [2]. The free radicals induced lesions have been considered as one of the leading causes of cancer [3]. Attention of the advanced clinical investigators has been focused on the therapeutic measures of this disease. Various therapeutic strategies are followed for the treatment of cancer and chemotherapy has been considered as the most acceptable and positive prognostic therapeutic approach [4]. The drugs from natural sources being biodegradable are preferred over the synthetic ones due to their comparative safe and efficacious nature [5]. Several natural anticancer drugs are available in the market like etoposide, docetaxel, irinotecan, pacletaxel, topotecan, vincristine and vinblastine [6]. Various derivatives of natural anticancer drugs are also being synthesized and exploited against cancer [7]. The exploration of anticancer agent is not confined to the laboratory rather their availability is also evidenced in plants, marine animals, bacteria, algae, fungi, reptiles etc [8, 9]. The most feasible and economic source of anticancer agents is plants. Numerous anticancer compounds have been isolated from plants and various investigators have reported plethora of plants’ secondary metabolites with strong anticancer potentials [10]. Several families of plants have been reported to possess anticancer compounds. One of the plants’ families i.e., Polygonaceae is also famous for anticancer activities [11]. Rumex is one of the most important genera of this family and several species of this genus have been reported to possess strong anticancer potentials [12]. Several antitumor compounds have also been isolated from different species of this genus, for example, *Rumex hymenosepalus* has been reported with the isolation of antitumor compounds, i.e. leucodelphinidin and leucopelargonidin [13]. Several species of Rumex have been employed ethnomedicinally in the treatment of inflammation, swelling, hyper proliferative skin diseases [14].

*Rumex hastatus* is one of the most important species which has been used traditionally for the treatment of various ailments like rheumatism, tonsillitis, piles etc [15–17]. Previously, the *R. hastatus* has been evaluated for anticancer potential against HepG2, MCF7 or LNCaP cell lines with considerable cytotoxicity [18]. Previously, *R. hastatus* has been evaluated for anticholinesterase, antioxidant, anti-tumor, anti-angiogenic, phytotoxic and antibacterial potentials [19–22]. Based on the ethnomedicinal uses and literature review of *R. hastatus*, the current study was designed to explore cytotoxic potential of this plant against cell lines and to find out the bioactive phytoconstituents responsible for anticancer activity using GC-MS analysis.

## Methods

### Plant collection, extraction and fractionation

The aerial parts of mature plant of *R. hastatus* were collected from the surrounding area of University of Malakand, Pakistan. The plant’s name was confirmed by Dr. Ali Hazrat, Plant Taxonomist, Department of Botany, Shaheed Benazir Bhutto University, Sheringal Dir (U), KPK, Pakistan, and deposited with voucher specimen No. 1015SA. The plant’s material was shade dried, powdered and subjected to maceration process. Afterwards, it was filtered and the filtrate was evaporated under reduced pressure using rotary evaporator at 40 °C [23, 24]. Similarly, the crude methanolic extract (Rh.Cr) was obtained weighing 400 g (5.7 %). The suspension of Rh.Cr weighing 300 g was subjected to fractionation process with the order of increasing polarity. In this way, the fractions obtained were 19 (6.3 %), 21 (7 %), 29 (9.6 %) and 120 (40 %) g of *n*-hexane (Rh.Hex), chloroform (Rh.Chf), ethyl acetate (Rh.EtAc) and aqueous fraction (Rh.Aq) respectively [25, 26].

### Gas Chromatography (GC) analysis

Samples were subjected to GC analysis using an Agilent USB-393752 gas chromatograph (Agilent Technologies, Palo Alto, CA, USA) with HHP-5MS 5 % phenylmethylsiloxane capillary column (30 m × 0.25 mm × 0.25 μm film thickness; Restek, Bellefonte, PA) equipped with an FID detector. The initial temperature of the oven was retain at 70 °C for 1 min, followed by increase at the rate of 6 °C/min to 180 °C for 5 min and finally at the rate of 5 °C/min to 280 °C for 20 min. The temperature of injector and detector were set at 220 and 290 °C, correspondingly. Helium was used as carrier gas at a flow rate of 1 ml/min, and diluted samples (1/1000 in *n*-pentane, v/v) of 1.0 μl were injected manually in the splitless mode.

### Gas Chromatography–Mass Spectrometry (GC/MS) analysis

GC/MS analysis of samples were processed using an Agilent USB-393752 gas chromatograph (Agilent Technologies, Palo Alto, CA, USA) with a HHP-5MS 5 % phenylmethylsiloxane capillary column (30 m × 0.25 mm × 0.25 μm film thickness; Restek, Bellefonte, PA) outfitted with an Agilent HP-5973 mass selective detector in the electron impact mode (Ionization energy: 70 eV) working under the same experimental conditions as described for GC.

### Identification of components

Compounds were recognized by comparison of their retention times with those of authentic compounds in the literature under the same set of conditions. Further identification were done through the spectral data obtained from the Wiley and NIST libraries and further confirmed by comparisons of the fragmentation pattern of the mass spectra with data published in the literature [27, 28].

### MTT assay on HeLa and NIH/3T3 cell lines

Cytotoxic activity of various samples of *R. hastatus* was assayed in 96-well flat-bottomed micro plates following the standard MTT (3-[4, 5-dimethylthiazole-2-yl]-2, 5-diphenyl-tetrazolium bromide) colorimetric assay [29]. Briefly, HeLa cells (Cervical Cancer) and Mouse embryonic fibroblast NIH/3T3 cell lines were cultured in Minimum Essential Medium Eagle. The media was supplemented with 5 % of fetal bovine serum (FBS), 100 μg/ml of streptomycin and 100 IU/ml of penicillin in 75 cm^2^ flasks and incubated in 5 % CO_2_ incubator at 37 °C. Growing cells were harvested exponentially and counted with haemocytometer followed by dilution with a particular medium. Cell culture was prepared having the concentration of 6 x 10^4^ cells/ml and transferred (100 μl/well) into 96-well plates. After overnight incubation, medium was discarded and 200 μl of fresh medium was added with various concentrations of plant samples (1–30 μM). After 48 h, 200 μl MTT (0.5 mg/ml) was added to each well and incubated additionally for 4 h. Afterward, 100 μL of DMSO was added to each well. The extent of MTT reduction to formazan within cells was figured out by measuring the absorbance at 570 nm, employing a micro plate reader (Spectra Max plus, Molecular Devices, CA, USA). The samples causing 50 % growth inhibition for both cell lines were recorded as IC_50_. The percent inhibition was calculated by the formula given below;

$$ \%\ \mathrm{Inhibition} = 100-\frac{\mathrm{Mean}\ \mathrm{O}\mathrm{D}\ \mathrm{of}\ \mathrm{test}\ \mathrm{sample}-\mathrm{Mean}\ \mathrm{O}\mathrm{D}\ \mathrm{of}\ \mathrm{negative}\ \mathrm{control}}{\mathrm{Mean}\ \mathrm{O}\mathrm{D}\ \mathrm{of}\ \mathrm{positive}\ \mathrm{control}-\mathrm{Mean}\ \mathrm{O}\mathrm{D}\ \mathrm{of}\ \mathrm{negative}\ \mathrm{control}} \times 100 $$

The results i.e., Percent inhibition were processed via Soft- Max Pro software (Molecular Device, USA).

### Statistical analysis

All the tests were performed in triplicate and values were expressed as means ± S.E.M. Multiple group comparison was performed by Two way ANOVA followed by Bonferroni post test in which the *P* < 0.05 were considered significant.

## Results

### MTT assays

The MTT assay was carried out against two types of cell lines, i.e., HeLa and NIH/3T3. The crude methanolic extract and sub-fractions of *R. hastatus* were assay against both cell lines. All the samples were found active against both cell lines with chloroform fraction more dominant as shown in Table 1. In HeLa cell line cytotoxicity assay, the chloroform fraction revealed significant cytotoxic potential. The observed cytotoxic potential against HeLe cell line were 81.50 ± 0.86, 69.00 ± 2.80, 43.66 ± 0.89 and 34.22 ± 0.23 % at concentrations of 500, 250, 125 and 62.5 μg/ml respectively with IC_50_ value of 151.52 μg/ml. Similarly, the second highest activity has been demonstrated by ethyl acetate fraction i.e., 79.66 ± 0.89, 66.32 ± 1.30, 40.93 ± 0.49 and 29.83 ± 1.36 % cytotoxic activity at concentrations of 500, 250, 125 and 62.5 μg/ml against HeLa cell line with IC_50_ value of 166.50 μg/ml. The methanolic extract and aqueous fraction demonstrated moderate cytotoxic potentials with IC_50_ values of 347.33 and 369.68 μg/ml respectively. Among all the samples of *R. hastatus*, the least activity was shown by that of *n*-hexane fraction with IC_50_ of 572.61 μg/ml.

**Table 1:** Cytotoxic activity of various samples of *Rumex hastatus* against HeLa and NIH/3T3 cell lines

Samples	Conc. (μg/ml)	HeLa Cell Line		NIH/3T3 Cell Line	
		Inhibition (%)	IC_50_ (μg/ml)	Inhibition (%)	IC_50_ (μg/ml)
Rh.Cr	500	63.25 ± 0.20***	347.33	74.96 ± 0.21***	174.52
	250	41.43 ± 1.15***		59.46 ± 0.54***	
	125	29.00 ± 1.50***		43.07 ± 1.02***	
	62.5	20.64 ± 1.60***		35.53 ± 0.61***	
Rh.Hex	500	36.33 ± 3.50***	572.61	53.86 ± 0.85***	439.26
	250	15.46 ± 2.43***		40.60 ± 0.41***	
	125	07.33 ± 0.68***		28.33 ± 0.33***	
	62.5	05.03 ± 0.23***		21.50 ± 0.60***	
Rh.Chf	500	81.50 ± 0.86***	151.52	82.13 ± 0.88***	53.37
	250	69.00 ± 2.80***		70.66 ± 0.49***	
	125	43.66 ± 0.89***		64.02 ± 1.11***	
	62.5	34.22 ± 0.23***		51.43 ± 0.61***	
Rh.EtAc	500	79.66 ± 0.89***	166.50	72.76 ± 0.78***	158.73
	250	66.32 ± 1.30***		59.00 ± 0.57***	
	125	40.93 ± 0.49***		46.86 ± 0.85***	
	62.5	29.83 ± 1.36***		31.43 ± 0.81***	
Rh.Aq	500	60.83 ± 1.36***	369.68	65.60 ± 0.41***	237.62
	250	42.53 ± 0.46***		51.96 ± 0.21***	
	125	33.61 ± 1.70***		42.66 ± 0.49***	
	62.5	21.33 ± 0.33***		36.13 ± 0.88***	
Doxorubicin	500	96.63 ± 1.67	<0.1	98.53 ± 1.09	<0.1
	250	91.87 ± 0.25		93.76 ± 0.78	
	125	89.46 ± 2.43		90.33 ± 0.88	
	62.5	84.50 ± 0.86		87.46 ± 0.54	

Data is represented as mean ± S.E.M; *n* = 3, ***: *P* < 0.001

*Key*: *Rh.Cr* Crude methanolic extract, *Rh.Hex n*-hexane fraction, *Rh.Chf* chloroform fraction, *Rh.EtAc* ethyl acetate fraction, *Rh.Aq* aqueous fraction

In NIH/3T3 cell line assay, again the chloroform fraction was found dominant exhibiting 82.13 ± 0.88, 70.66 ± 0.49, 64.02 ± 1.11 and 51.43 ± 0.61 % cytotoxic potential at concentrations of 500, 250, 125 and 62.5 μg/ml with IC_50_ value of 53.37 μg/ml. Similarly, the ethyl acetate fraction revealed the second highest activity against NIH/3T3 cell line i.e., 72.76 ± 0.78, 59.00 ± 0.57, 46.86 ± 0.85 and 31.43 ± 0.81 % at concentrations of 500, 250, 125 and 62.5 μg/ml with IC_50_ value of 158.73 μg/ml. The IC_50_ calculated for the rest of the samples were 174.52, 237.62 and 439.26 μg/ml for methanolic extract, aqueous and *n*-hexane fractions respectively. The cytotoxic potential of all the test samples of *R. hastatus* against NIH/3T3 cell line has been summarized in Table 1. The standard drug doxorubicin exhibited IC_50_ value <0.1 μg/ml against both cell lines.

### GC-MS analysis

Based on the high potency in both cell lines assays, the chloroform fraction was subjected to GC-MS analysis. A total of 78 phytoconstituents were identified by the GC-MS analysis. The identified compounds contain important bioactive compounds responsible for the cytotoxic potential of the plant. The parameters of some compounds found in GC-MS analysis have been summarized in the Table 2.

**Table 2:** Parameters of various components in Chloroform fraction of *Rumex hastatus*

RT (min)	Height	Height %	Area	Area %	Area Sum %	Base Peak m/z	Width
26.577	536469	7.54	1916591	8.14	2.26	222	0.144
28.475	6E + 06	87.45	22348531	94.9	26.38	88	0.204
31.979	7E + 06	91.91	22675632	96.29	26.77	67.1	0.141
32.106	7E + 06	100	23550533	100	27.8	55.1	0.127
32.173	333815	4.69	496177	2.11	0.59	55.1	0.054
32.525	900308	12.66	2370371	10.07	2.8	88	0.107
34.939	467634	6.58	1286192	5.46	1.52	254	0.1
35.766	331299	4.66	836122	3.55	0.99	88	0.097
37.977	340828	4.79	773168	3.28	0.91	149	0.09
43.667	851097	11.97	2994991	12.72	3.54	43.2	0.134

It is evident that area wise the highest percentage has been exhibited by linoleic acid ethyl ester with retention time 31.979 (96.29 %) followed by hexadecanoic acid, ethyl ester with retention time 28.475 (94.9 %). A summary of all identified compounds in the chloroform fraction has been shown in Table 3.

**Table 3:** List of compounds in chloroform fraction of *Rumex hastatus*

S. No	Compound Label	RT	Common Name	Formula	Hits (DB)
1.	Diethyl 2,2-Dihydroxy Sulfide	5.757	Tedegyl	C4H10O2S	3
2.	Benzenemethanol	6.438	Benzyl alcohol	C7H8O	10
3.	2-Pyrrolidinone, 1-methyl	6.567	M-Pyrol	C5H9NO	10
4.	4H-Pyran-4-one, 2,3-dihydro-3,5-dihydroxy-6-methyl-	8.793	NF	C6H8O4	10
5.	Benzoic acid, ammonium salt	9.343	Ammonium benzoate	C7H6O2	10
6.	2-Methoxy-4-vinylphenol	12.609	p-Vinylguaiacol	C9H10O2	10
7.	Trimethylsilyl cyanide	15.284	Trimethyl silyl nitrile	C4H9NSi	10
8.	Bis(2-hydroxyethyl)lauramide	17.708	lauramide	C16H33NO3	10
9.	Dodecanoic acid, ethyl ester	18.281	Ethyl dodecanoate	C14H28O2	10
10.	2-Cyclopenten-1-one, 3-methyl-2-pentyl	18.547	Dihydrojasmone	C11H18O	10
11.	Ethyl.alpha.-d-glucopyranoside	19.004	glucopyranoside	C8H16O6	10
12.	Silane, [(1,1-dimethyl-2 propenyl)oxy] dimethyl-	19.332	NF	C7H16OSi	10
13.	4-[1,5-Dimethyl-1,4-Hexadienyl]-1-Methyl-1-Cyclohexene	19.582	NF	C15H24	10
14.	Ar-tumerone	19.755	Ar-tumerone	C15H20O	10
15.	4-((1E)-3-Hydroxy-1-propenyl)-2-methoxyphenol	21.382	NF	C10H12O3	10
16.	Tetradecanoic acid	21.798	Myristic acid	C14H28O2	10
17.	(-)-Loliolide or Loliolide	22.21	Calendin	C11H16O3	10
18.	Tetradecanoic acid, ethyl ester	22.642	Ethyl myristate	C16H32O2	10
19.	2-Cyclohexen-1-one, 4-hydroxy-3,5,6-trimethyl-4-(3-oxo-1-butenyl)	22.779	NF	C13H18O3	10
20.	p-Hydroxycinnamic acid, ethyl ester	23.832	p-Hydroxycinnamic acid, ethyl ester	C11H12O3	10
21.	7,11,15-Trimethyl,3-Methylene-1-Hexadecene	24.028	Neophytadiene	C20H38	10
22.	2-Pentadecanone, 6,10,14-trimethyl	24.223	Hexahydrofarnesyl acetone	C18H36O	10
23.	Pentadecanoic acid, ethyl ester	25.763	ethyl pentadecanoate	C17H34O2	10
24.	Ethyl (2E)-3-(4-hydroxy-3-methoxyphenyl)-2-propenoate	26.577	NF	C12H14O4	6
25.	Hexadecanoic acid	27.756	Palmitic acid	C16H32O2	10
26.	Ethyl 9-Hexadecenoate	27.899	NF	C18H34O2	10
27.	1,9-Tetradecadiene	28.273	NF	C14H26	10
28.	Hexadecanoic acid, ethyl ester	28.475	Ethyl palmitate	C18H36O2	10
29.	(E)-3-(4-Biphenylyl)-2-propen-1-ol	28.518	NF	C15H14O	8
30.	Peniopholide	29.798	Peniopholide	C15H24O3	10
31.	Heptadecanoic acid, ethyl ester	30.025	Ethyl n-heptadecanoate	C19H38O2	10
32.	Propyl hexadecanoate	30.527	Propyl palmitate	C19H38O2	10
33.	Heptadecanoic acid, ethyl ester	30.607	Ethyl n-eptadecanoate	C19H38O2	10
34.	2-Hexadecen-1-ol, 3,7,11,15-tetramethyl-, [R-[R*,R*-(E)]]-	31.016	Phytol	C20H40O	10
35.	cis-9,cis-12-Octadecadienoic acid	31.507	NF	C18H32O2	10
36.	E-11,13-Tetradecadien-1-ol	31.616	NF	C14H26O	10
37.	Linoleic acid ethyl ester	31.979	Mandenol	C20H36O2	10
38.	Ethyl 9-Octadecanoate	32.104	Ethyl 9-Octadecenoate	C20H38O2	10
39.	exo-4-Methylbicyclo[3.2.1]octan-3-ene	32.121	NF	C9H14	10
40.	16-Methyloxacyclohexadeca-3,5-dien-2-one	33.111	NF	C16H26O2	10
41.	3.beta.-Hydroxydihydroconfertifolin	33.956	NF	C15H24O3	1
42.	Ethyl 9-Hexadecenoate	34.021	NF	C18H34O2	10
43.	Cis-8-methyl-exo-tricyclo[5.2.1.0(2.6)]decane	34.647	NF	C11H18	10
44.	9,10-Anthracenedione, 1,8-dihydroxy-3-methyl	34.942	C.I. Natural Yellow 23	C15H10O4	10
45.	4,8,12-Trimethyltridecan-4-olide	35.181	NF	C16H30O2	10
46.	5-Icosyne	35.305	5-Eicosyne	C20H38	10
47.	Ethyl 9-Hexadecenoate	35.382	NF	C18H34O2	10
48.	Heptadecanoic acid, ethyl ester	35.768	NF	C19H38O2	10
49.	13-Tetradecenal	35.985	NF	C14H26O	10
50.	5-Dodecyne	36.078	5-Dodecyne	C12H22	10
51.	N-Vanillylnonanoamide	37.013	Nonivamide	C17H27NO3	10
52.	1,2-Benzenedicarboxylic acid, bis (2 ethylhexyl) ester	37.978	DNOP	C24H38O4	10
53.	N(4-Hydroxy-3-Methoxybenzyl)-8-Methylnon-6-Enamide	38.186	NF	C18H27NO3	10
54.	delta.13-cis-Docosenoic acid	38.242	Erucic acid	C22H42O2	10
55.	N-(4-Hydroxy-3-Methoxybenzyl)-8-Methyl-Nonanamide	38.489	NF	C18H29NO3	10
56.	Docosanoic acid, ethyl ester	38.566	Ethyl docosanoate	C24H48O2	10
57.	9,10-Anthracenedione, 1,8-dihydroxy-3-methoxy-6-methyl	39.322	Physcion	C16H12O5	10
58.	Methyl palustrate isomer	39.554	Methyl palustrate	C21H32O2	1
59.	1-Bromo-4,8,12-trimethyl-3(E),7(E)-11-tridecatriene	40.642	NF	C16H27Br	5
60.	Oleic acid amide	40.909	Oleamide	C18H35NO	10
61.	Heptadecanoic acid, ethyl ester	41.07	NF	C19H38O2	10
62.	1,1-Di(1,1-dimethylethyl)cyclopropane	41.672	NF	C11H22	3
63.	Arachic alcohol	41.685	n-Eicosanol	C20H42O	10
64.	Aristol-9-en-8-one	42.368	Aristolone	C15H22O	10
65.	2-Bromotetradecane	42.397	NF	C14H29Br	10
66.	Stigmasta-5,22-dien-3-ol, acetate, (3.beta.,22Z)-	42.529	NF	C31H50O2	10
67.	Stigmast-5-en-3-ol, (3.beta.,24S)- (CAS)	42.968	Clionasterol	C29H50O	10
68.	7-methyltocol	43.226	NF	C27H46O2	2
69.	Stigmast-5-en-3-ol, acetate, (3.beta.)-	43.666	β-Sitosterol acetate	C31H52O2	10
70.	alpha.-Tocopherol	44.466	Vitamin E	C29H50O2	7
71.	Cholesta-4,6-dien-3-ol, benzoate, (3.beta.)	45.533	NF	C34H48O2	9
72.	Alpha.-Bisabolol	52.989	.Alpha.-bisabolol	C18H32O	10
73.	Methyl Commate E	53.773	NF	C31H50O5	10
74.	Stigmast-4-en-3-one	55.721	Sitostenone	C29H48O	10
75.	2-Ethylthio-2-ethoxy-3-oxo-N phenylbutanamide	57.414	NF	C14H19NO3S	9
76.	3-(Methoxymethoxy)-5-(phenylmethoxypentanal	58.739	NF	C14H20O4	1
77.	13-Epimanool	62.472	Epimanool-	C20H34O	10
78.	1,2-Dicyclohexyl-1,1,2,2-tetrafluoroethane	70.638	NF	C14H22F4	6

**Fig. 1:**
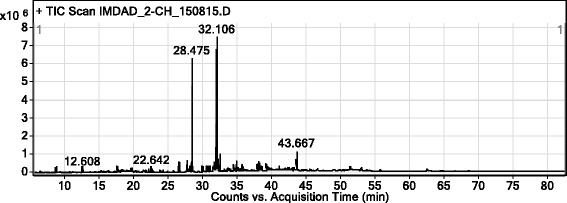
GC-MS chromatogram of chloroform fraction of *Rumex hastatus*

**Fig. 2:**
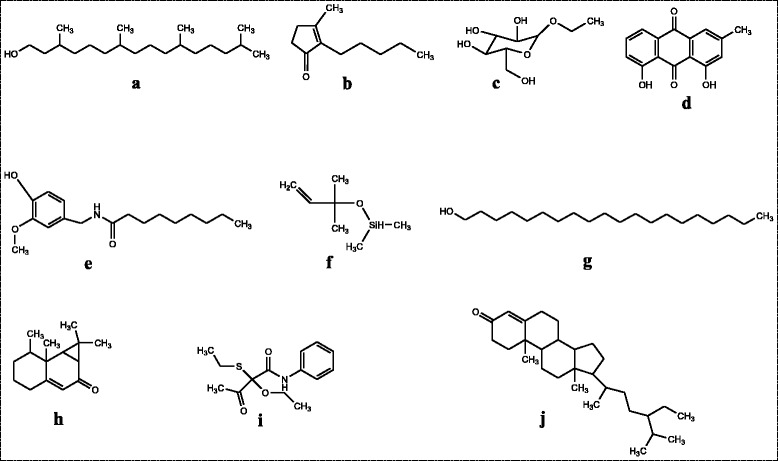
Structures of some anticancer compounds identified in the GC-MS analysis of chloroform fraction of *Rumex hastatus.* **a** Phytol **b** Dihydrojasmone **c** Ethyl.alpha.-d-glucopyranoside **d** Anthracenedione **e** Nonivamide **f** Silane **g** Eicosanol **h** Aristolone **i** 2-Ethylthio-2-ethoxy-3-oxo-N-phenylbutanamide and **j** Sitostenone

**Fig. 3:**
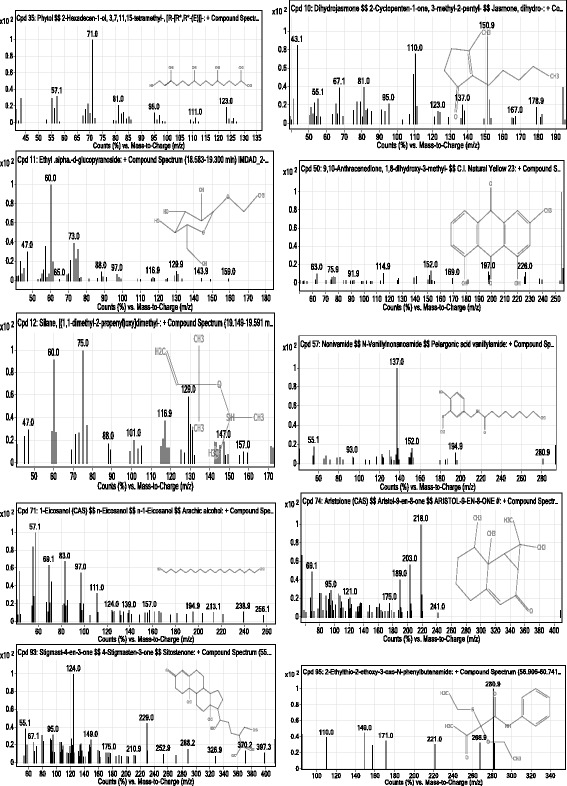
GC-MS spectra of some important compounds in chloroform fraction of *Rumex hastatus*

The GC-MS chromatogram of the chloroform fraction is shown in Fig. 1 in which some of the important peaks are clearly visible. Some important bioactive compounds which having a positive role in cytotoxicity are sorted in Fig. 2. Moreover, the integration patterns of some important compounds as elucidated by GC-MS are shown in Fig. 3.

## Discussion

HeLa is a type of immortal cell line obtained from cervical cancer cells and for the very first time this cell line has been taken from late Henrietta Lacks in 1951 and abbreviated for her name [30]. Similarly, the NIH/3T3 cell line was originated from swiss mice in 1962 which consists of immortal fibroblast cell and widely used for experimental purposes [31]. To figure out the cytotoxicity in these cells, the MTT assay is considered as a rapid and authentic procedure to appraise the cell viability and death by calorimetric analysis [29]. Previously, the MTT assay has been reported by numerous researchers to evaluate the cytotoxicity [32, 33]. Recently, *Polygonum hydropiper* has been demonstrated with significant cytotoxicity against NIH/3T3 cell line following MTT assay [34]. As this is evidenced from several reports that a specific pharmacological potential within plant species is basically conferred due to specific group of compounds [35]. Similarly, a specific group of phytoconstituents is responsible for the cytotoxic potential of certain plants [36]. The GC-MS is a quick and easy way of finding out various components in a crude mixture of plant extract [37]. In our current research, the GC-MS analysis of chloroform fraction of *R. hastatus* showed 78 compounds summarized in Table 2. Several compounds identified by GC-MS in the chloroform fraction are reported to have positive role in cell toxicities. For instance, phytol, dihydrojasmone, ethyl α-d-glucopyranoside, anthracenedione, silane, nonivamide, eicosanol, aristolone, ar-tumerone and sitostenone are the compounds with cytotoxic/anticancer potential demonstrated along with their spectra in Figs. 2 and 3.

**Fig. 4:**
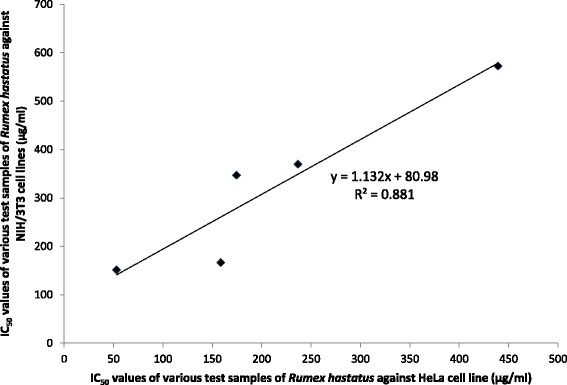
Regression and correlation of various samples of *Rumex hastatus* against HeLa cell line Vs NIH/3T3 cell line

Phytol present in *R. hastatus* has been reported to induce programmed cell death in human lymphoid leukemia Molt 4B cells [38]. Dihydrojasmone, one of the member of jasmonate family, which has been implied as a new family of anticancer agents [39]. Ethyl-α-d-glucopyranoside a derivative of glucopyranoside has been reported time and again to possess strong anticancer potential and it is evident from the GC-MS analysis that *R. hastatus* contain ethyl α-d-glucopyranoside, which may confer the possible anticancer potential to this plant. Anthracenedione has also been reported to possess anticancer properties [40]. Silane has been proven as an efficient agent in a nanoparticle based drug delivery system for anticancer compounds. The chloroform fraction of *R. hastatus* also possess nonivamide, which is skin permeation enhancer and used in various ointments etc [41]. Similarly, eicosanol is a C_20_ alcohol present in *R. hastatus* and C_20_ aliphatic alcohols has been employed in the treatment of hyperproliferative skin disordersone [42]. Aristolone and Ar-tumerone are sesquiterpenes, and the derivatives of sesquiterpene have been reported to possess the cytotoxic potential [43]. Likewise, vitamin E a phenolic compound with pronounced free radical scavenging and anticancer potential has also been evidenced from Table 2 [44, 45]. Another compound i.e., a natural steroid named sitostenone has also been analyzed in GC-MS spectra and steroids have also been used since long for the treatment of cancer, so this compound may also be involved in cytotoxicity observed in our current studies [46]. The current investigational study demonstrates that the chloroform fraction of *R. hastatus* was the most active one against two types of cell lines. The regression and correlation analysis shows that this plant has a parallel cytotoxic potential against both the cell lines as depicted in the Fig. 4 with r^2^ value of 0.881. The current study can also be correlated with the previous cytotoxic activity of *R. hastatus* against brine shrimps in which the chloroform fraction was the most active fraction [22]. Based on the marked potential of this fraction, it has been chemically characterized and based on the literature survey; the active compounds have been sorted out.

## Conclusion

Based on our current results, we can conclude that *Rumex hastatus* is a potential source of cytotoxic compounds. Moreover, the chloroform fraction is the active one among other solvent fractions of *R. hastatus*. Based on the GC-MS analysis of chloroform fraction, we can conclude that the chloroform fraction of *R. hastatus* is a rich source of bioactive compounds responsible for cytotoxicity.

## Abbreviations

eV: Electron volt
FBS: Fetal bovine serum
FID: Flame ionization detector
GC-MS: Gas chromatography-mass spectrometry
HeLa: Human cervical carcinoma cell line or Henrietta Lacks cell line
HepG2: Human liver cancer cell line/Hepatoblastoma G2 cell line
IC_50_: Median inhibitory concentration
LNCaP: Lymph node carcinoma of the prostate
MTT: 3-[4, 5-dimethylthiazole-2-yl]-2, 5-diphenyl-tetrazolium bromide
MCF7: Breast cancer cell line/Michigan Cancer Foundation-7
NIH/3T3: Fibroblast cell line from Swiss mouse embryo/3-day transfer, inoculum 3 x 10^5^ cells
NIST: National Institute of Standards and Technology
OD: Optical density
Rh.Aq: Aqueous fraction
Rh.Chf: Chloroform fraction
Rh.Cr: Methanolic extract of Rumex hastatus
Rh.EtAc: Ethyl acetate fraction
Rh.Hex: n-hexane fraction
SEM: Standard error mean
